# Does Problematic Use of Social Network Mediate the Association between Bullying Victimization and Loneliness among Lebanese Adolescents?

**DOI:** 10.3390/children10030599

**Published:** 2023-03-21

**Authors:** Elia Eid, Feten Fekih-Romdhane, Abir Sarray El Dine, Diana Malaeb, Souheil Hallit, Sahar Obeid

**Affiliations:** 1 Research Department, Psychiatric Hospital of the Cross, Jal Eddib P.O. Box 60096, Lebanon; 2 Faculty of Medicine of Tunis, Tunis El Manar University, Tunis 1068, Tunisia; 3 Department of Psychiatry “Ibn Omrane”, The Tunisian Center of Early Intervention in Psychosis, Razi Hospital, Manouba 2010, Tunisia; 4 Department of Biomedical Sciences, School of Arts and Sciences, Lebanese International University, Mazraa, Beirut P.O. Box 146404, Lebanon; 5 School of Pharmacy, Gulf Medical University, Ajman P.O. Box 4184, United Arab Emirates; 6 School of Pharmacy, Lebanese International University, Mazraa, Beirut P.O. Box 146404, Lebanon; 7 School of Medicine and Medical Sciences, Holy Spirit University of Kaslik, Jounieh P.O. Box 446, Lebanon; 8 Applied Science Research Center, Applied Science Private University, Amman 11937, Jordan; 9 Social and Education Sciences Department, School of Arts and Sciences, Lebanese American University, Jbeil P.O. Box 36, Lebanon

**Keywords:** problematic social network use, loneliness, bullying victimization, adolescents

## Abstract

(1) Background: Bullying victimization has been associated with several behavioral outcomes, particularly loneliness. Similarly, an increase in social network use has been identified in recent years, particularly during the COVID-19 pandemic, and has been shown to be associated with bullying and loneliness. Investigating the mediating factors of loneliness among bullied adolescents is useful for taking preventive measures in the Lebanese population. This study aims to examine the association between bullying victimization and loneliness among Lebanese adolescents while considering the indirect effect of problematic social network use. (2) Methods: We carried out a cross-sectional study, between January and April 2022, that enrolled 379 adolescent Lebanese students (64.9% females, mean age 16.07 ± 1.19 years) who were current residents of Lebanon (15 to 18 years), and were from the five governorates of Lebanon (Beirut, Mount Lebanon, North, South and Bekaa). The snowball method was applied to select our sample; an electronic copy of the questionnaire was created using the Google Forms software and an online strategy was designed to collect the data. (3) Results: Negative social comparison and addictive consequences of problematic use of social network mediated the association between bullying victimization and loneliness. Higher bullying victimization was significantly associated with higher negative social comparison and addictive consequences of problematic use of social network, which in turn were significantly associated with more loneliness. Finally, higher bullying victimization was directly significantly associated with more loneliness. (4) Conclusions: Studying the mediating factors of loneliness in bullied adolescents can improve our understanding of this topic, allowing us to propose new interventions to prevent psychological problems in adolescents. Future studies are needed to further clarify the physiological processes that underlie the associations between social triggers and loneliness during adolescence.

## 1. Introduction

Adolescence is considered a critical stage marked by transitions in behavior and mental and physical states [1]. During this age, complex changes occur in the human brain [2]. Throughout adolescence, many factors tend to combine, making this phase of development vulnerable to stressors, particularly in terms of neurobiological processes [3]. Therefore, stressful experiences at this critical period of development can alter the maturation process of the brain and may lead to an increase in psychological morbidities, commonly noticed during adolescence [3].

Social relationships significantly affect our daily psychosocial development, in particular in adolescence. The COVID-19 pandemic has caused a significant shift in how we arrange ourselves as social beings, particularly among youth who have experienced a withdrawal from school and activities [4]. Therefore, while the implementation of restrictions during the COVID-19 period has reduced the transmission of the virus, this stay-at-home period has led to reduced human contact, and thus loneliness [5]. Loneliness can be defined as a stressful emotion caused by the feeling that a person’s social demands are not needs are not met by the quantity or quality of personal connections [6,7]. Several researchers consider that most people, at some stage in their lives, have experienced loneliness [8,9]. Currently, research is focusing on factors linked to loneliness. In fact, loneliness seems to be associated with several behavioral and emotional factors, such as bullying victimization (BV) [10,11], negative childhood experiences [12], and lack of personal companions [12]. Various sociodemographic factors are additionally associated with the level of loneliness [13,14]. Being female (compared to male), having secondary education level, and lower monthly income were found to be linked to a greater sense of loneliness [15]. In addition, age has been found to be associated with loneliness [16,17]. Studies have found that younger participants have higher levels of loneliness [16,17].

BV is a source of psychosocial stress characterized by an inequality of power between the victim and the bully [18,19]. Many studies have shown that BV can negatively affect adolescents’ mental health and well-being [20,21]. Between 2003 and 2015, BV was reported by 30.5% of teenagers aged between 12 and 17 years [22]. Studies have shown that the most affected regions were The Eastern Mediterranean Region (45.1%) and African Region (43.5%), whereas Europe (8.4%) had the lowest prevalence [22]. In Lebanon, bullying affects 25% of Lebanese adolescents, with 90% of incidences occurring in schools [23,24]. The level of BV was found to be higher in male gender, lower socioeconomic status, and younger age [22]. One of the implications caused by BV is loneliness, as many studies relate the development of loneliness to factors such as BV [25,26]. At last, bullying was associated with higher levels of internalizing problem behaviors such as loneliness as well as higher levels of externalizing problem behaviors such as addiction [26].

On the other hand, an increase in the use of social networks has been identified in recent years [27], especially during the COVID-19 pandemic [28]. The population, especially young people have switched to social media in order to compensate for the loss of social support faced, particularly during lockdowns [16]. Since the beginning of the pandemic, Facebook reported an increase of 70% in the number of hours spent on its social media network and an increase of more than 50% in time spent on chatting [28]. This has led to an increase of Problematic Use of Social Networking (PUSN) [16,29]. PUSN is described as a lack of control over social media use, which can affect their daily life and increase their feelings of distress [30,31]. According to a Lebanese study, 3.6% of the study population of Lebanese adolescents were found to have a high level of PUSN, whereas 40% of the sample were found to be occasional or frequent Internet users [32].

Beside addictive consequences, a key factor in PUSN, known as negative social comparison [33], is studied as the second component of the PUSN scale [34]. Social comparison is defined as a self-evaluation where the person compares themselves to others [35]. On the other hand, social comparison (SC) was found to be associated to bullying [36]. A study found that attributions of SC can predict how children and adolescents respond when bullied [36]. The SC direction reflects the children’s efficacy in dealing with victimization [36]. Moreover, SC is considered to be an important factor linked to social network use and loneliness [37]. People tend to consider the people they see on social networks are happier and have a more exciting life than they do [38,39]. According to a research of a sample of Facebook users, those who compare themselves to their Facebook friends are more prone to experience loneliness [37].

Finally, the use of social networking sites has been linked to an increase in psychosocial problems, including loneliness [16,40]. On the other hand, previous studies have found that lonely people use the Internet to avoid loneliness [41]. Moreover, PUSN has been found to be linked with BV. While BV has been associated with several types of addiction such as nicotine and alcohol dependence, it has also been linked to PUSN as a coping mechanism for dealing with stress and seeking comfort and support in digital world [42,43,44].

**Figure 1:**
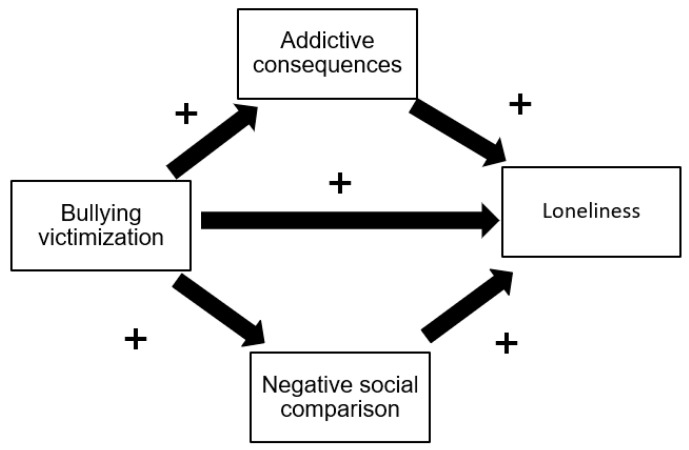
A conceptual framework illustrating the pathways from exposure (bullying victimization) to outcome (loneliness [10]) through mediators (addictive consequences [16,42,44] and negative social comparison [36,37]).

The above studies have shown that researchers have long considered BV, PUSN, and loneliness to be major public health issues, in particular in adolescence, and that there is at least a direct or indirect relationship between these problems (BV and PUSN) and the experience of loneliness. However, there is insufficient evidence of the direction and strength of these associations. Furthermore, with public protests, the COVID-19 pandemic, the Beirut blast, financial and social issues, the increase in child labor and child marriage, physical and mental health problems are expected to worsen among Lebanon’s children and adolescents [45,46]. Therefore, our research aims to investigate the association between bullying victimization and loneliness among Lebanese adolescents while considering the indirect effect of problematic social network use (see Figure 1).

## 2. Materials and Methods

### 2.1. Study Design and Participants

We conducted a cross-sectional study between January and April 2022 and included 379 adolescents. Inclusion criteria were community adolescents aged 13–17 years currently residing in Lebanon, and from one of the five Lebanese governorates (Beirut, Mount Lebanon, North, South, and Bekaa). We used the snowball sampling method to collect the data. Using Google Forms software, we created an online copy of the questionnaire and an online data collection method was used. We provided participants with the main objectives and goals of the study online prior to their participation, as well as instructions for completing the questionnaire. Afterwards, the first respondents were invited to send the questionnaire to other participants whom they knew and who would meet the age criteria of the study and be as diverse as possible in terms of location within the Lebanese governorates. There were no credits for participation.

### 2.2. Minimal Sample Size Calculation

Using the formula proposed by Fritz and MacKinnon [47], a minimum sample size of 127 was considered to be required for sample size estimation: $n=\frac{L}{f2}+k+1$. We considered *f* = 0.26 for a small to moderate effect size with *L* = 7.85 for an α error of 5% and power β = 80%. Ten variables were included in the model.

### 2.3. Questionnaire

The first section of the questionnaire included a description of the topic and purpose of the study, a confidentiality statement for respondents, and an instruction for the student to obtain parental permission before participating. The student was required to check the box “I got my parents’ approval and I consent to participate in this study” to complete the questionnaire.

The second section of the questionnaire included sociodemographic data (age, gender, governorate, self-reported current weight, and height). The Body Mass Index (BMI) was determined in accordance with World Health Organization guidelines [48]. The Household Crowding Index (HCI), which reflects the socioeconomic status of the family [49], is the ratio of the number of household occupants to the number of rooms in the house (excluding the kitchen and bathroom). The physical activity index is a measure of physical activity that takes into account the intensity, duration, and frequency of daily activity [50]. Regarding financial burden, participants were asked “How much pressure do you feel with regard to your personal financial situation in general?” on a scale of 1 to 10, with 10 indicating overwhelming financial pressure.

The third section consisted:

- The Illinois Bully scale (IBS). The IBS, validated in Lebanon [51], is an eighteen-item scale consisting of two subscales: bullying perpetration (e.g., “I annoyed other students”) and bullying victimization (e.g., “Other students beat and pushed me”) [52]. Each item was scored as follows: 0 for never and 4 for up to seven or more times. The respective items are added up to obtain the subscale scores. Higher scores for victimization and perpetration of bullying indicated, respectively, greater victimization and greater perpetration of bullying [53]. In our study, we used only the bullying victimization subscale (α = 0.88).
- Problematic Use of SNs (PUS) Scale. The PUS is a self-report scale that examines the potential impact of social networking’s (SNs) use on addiction, with a focus on the way SNs are being used in a comparative manner, and evaluating problematic SN use without a specific focus on a particular social media network, and therefore can be generalized to various SNs [34]. The instrument is composed of 18 Likert-type items with five possible options (1 = completely disagree, 5 = completely agree). Items are divided into two subscales: 8 items for addictive consequences (e.g., using social networks, I lose track of time and ignore important tasks I have outstanding) and 10 items for negative social comparison (e.g., when I see content from influencers or celebrities, I feel inferior). Higher scores reflecting higher problematic use of SNs (α = 0.95 for negative social comparison and α = 0.90 for addictive consequences).
- Jong-Gierveld Loneliness Scale. In our study, we used the modified 5-item version of the Jong-Gierveld Loneliness Scale [54], this scale assesses subjective loneliness. (e.g., I experience a general sense of emptiness; I miss having people around). Items were rated according to a yes/no type of answer; one point was awarded for a positive response and no points for a negative response. Higher scores reflect greater loneliness (α = 0.76). This scale is validated in Lebanon [55].

### 2.4. Translation Procedure

Forward and backward translation methods were applied to the PUS. A Lebanese translator who was not involved in the study translated the English version into Arabic. Then, the Arabic version was then translated back into English by a Lebanese psychologist proficient in English. Both versions, the original and the second English version, were checked for inconsistencies.

### 2.5. Statistical Analysis

Data analysis was performed using SPSS version 23 software. For each scale and subscale, Cronbach’s alpha values were calculated. Since all questions were required, we did not obtain any missing data. For reliability tests of all scales and subscales, we reported Cronbach’s alpha values. The loneliness score had a normal distribution, with skewness and kurtosis ranging from −1 to +1 [56]. We used Student’s *t* test and ANOVA to compare two and three or more means, respectively. To compare two continuous variables, we used Pearson’s correlation test. We used The PROCESS SPSS Macro version 3.4, model four [57] to calculate three pathways. Pathway A identified the regression coefficient for the effect of bullying victimization on problematic social network use; the study of the association between problematic social network use and loneliness score was shown by pathway B, and Pathway C’ was calculated to estimate the direct effect of victimization on the loneliness score. For indirect effects, if the bootstrapped 95% confidence intervals of the indirect AB pathway did not cross zero, an indirect effect was considered significant. We entered into the multivariable and mediation models whose variables showed a *p* < 0.25 in the bivariate analysis. We fixed the level of significance at *p* < 0.05.

## 3. Results

### 3.1. Sociodemographic and Other Characteristics of the Participants

This study included a total of 379 adolescents, with a mean age of 16.07 ± 1.19 years, and 64.9% females. Other characteristics are summarized in Table 1.

### 3.2. Bivariate Analysis

A higher mean loneliness score was seen in females compared to males (2.11 ± 1.69 vs. 1.83 ± 1.79; *p* = 0.124), with the difference being non-significant. Higher negative social comparison (*r* = 0.52), addictive consequences of problematic use of social network (*r* = 0.42), more bullying victimization (*r* = 0.27), older age (*r* = 0.14), and more financial burden (*r* = 0.25) were significantly associated with more loneliness. The bivariate analysis results are shown in Table 2.

### 3.3. Indirect Effect Analysis

**Figure 2:**
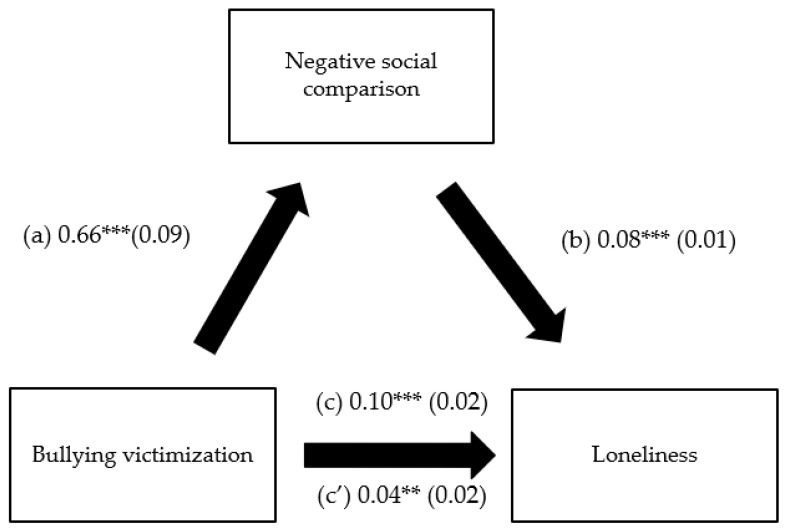
(**a**) Relation between bullying victimization and negative social comparison (R^2^ = 0.160); (**b**) Relation between negative social comparison and loneliness (R^2^ = 0.328); (**c**) total effect of the relation between bullying victimization and loneliness (R^2^ = 0.154); (**c’**) direct effect of the relation between bullying victimization and loneliness. Numbers are displayed as regression coefficients (standard error). *** *p* < 0.001; ** *p* < 0.01.

**Figure 3:**
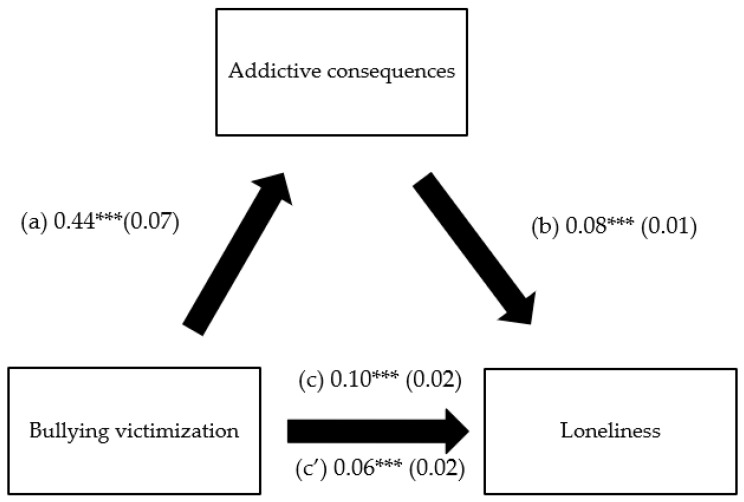
(**a**) Relation between bullying victimization and addictive consequences of problematic use of social network (R^2^ = 0.119); (**b**) relation between addictive consequences of problematic use of social network and loneliness (R^2^ = 0.254); (**c**) total effect of the relation between bullying victimization and loneliness (R^2^ = 0.153); (**c’**) direct effect of the relation between bullying victimization and loneliness. Numbers are displayed as regression coefficients (standard error). *** *p* < 0.001.

The results of the mediation analysis were adjusted over the following variables: age, sex, and financial burden. Negative social comparison and addictive consequences of problematic use of social network mediated the association between bullying victimization and loneliness (Table 3, Figure 2 and Figure 3). Higher bullying victimization was significantly associated with higher negative social comparison and addictive consequences of problematic use of social network, which in turn were significantly associated with more loneliness. Finally, higher bullying victimization was directly significantly associated with more loneliness.

## 4. Discussion

To our knowledge this is the first preliminary study to evaluate the association between BV and loneliness among Lebanese adolescents, taking into account the indirect effect of PUSN. These findings may contribute to the development of loneliness prevention strategies for future generations.

### 4.1. BV and Loneliness: Direct Effect

Our study showed a direct effect of BV on loneliness. Several studies have found that bullied people are more likely to experience physical health problems [58], emotional and behavioral problems [59], and mental disorders [60]. Our result is similar to other studies that have found that being bullied was connected to higher levels of loneliness [61,62]. In fact, studies have shown that bully victims are more likely to internalize problems such as loneliness [11,63]. In addition, stressful situations such as BV can cause feelings of social isolation accompanied by negative thoughts and loss of confidence in oneself [64]. As a result, challenges dealing with psychosocial stressors may cause reduced social connectedness, hence feeling of loneliness [64]. On the other hand, abusers may believe that their victims’ loneliness indicates their vulnerability to bullying, which may predispose lonely adolescents to frequent bullying [65,66]. Neurobiologically, BV is considered to be a severe uncontrollable psychological stress that triggers brain development and neurocognitive functioning in adolescents [20]. Furthermore, research has shown that loneliness can be induced by stress-induced biological factors [67]. Therefore, BV can induce loneliness directly through behavioral and neurobiological factors.

### 4.2. BV and PUSN

Consistent with previous studies [51,68,69], BV has been associated with PUSN. Several studies have identified BV as a factor associated with substance use [42,70,71]. According to research, children and adolescents who are bullied are more likely to use alcohol, tobacco, and illicit drugs [42,70,71]. Simultaneously, with the rise of technology, adolescents seek immediate rewards through social networks [72], especially during COVID-19 pandemic [73]. The use of social networks, in particular, might be considered as a coping method for escaping the interpersonal distress [74]. It has also been considered a psychosocial escape to avoid facing daily triggers, such as BV, in order to virtually construct an ideal life without stress and limitations [75].

### 4.3. PUSN and Loneliness

Throughout the lockdown, people turned to social media for social compensation, as PUSN was widespread in many countries [76]. This use of social networks increases the risk of PUSN and associated psychosocial problems [16]. Therefore, adolescents with excessive social media use exhibit an obsession and refusal to avoid using social media despite harmful consequences [77]. Research has shown that adolescents with PUSN usually develop social avoidance [78], in line with the result of our study stating that higher PUSN was significantly associated with more loneliness. Moreover, like other kinds of behavioral addiction, PUSN has been linked to interpersonal relationship problems [79] resulting in relationship dissatisfaction [80]. As a result, social media addiction contributes to increased mental health issues, notably depressive symptoms and loneliness [81]. In fact, previous research has shown that PUSN leads to loneliness [15,82]. One study showed that an increased social media use was associated with a higher feeling of loneliness among users [83]. According to research, the relationship between Internet addiction and other psychopathological symptoms is bidirectional [84]. In this line, PUSN is associated with social phobia and depression [85]. To cope with their social anxiety, lonely people may use the Internet to interact online without face-to-face interaction [86,87].

### 4.4. BV and Loneliness: The Indirect Role of PUSN

The results of the mediation analysis using PUSN as the mediating variable showed that PUSN fully mediated the association between BV and loneliness, with a R^2^ squared association index > 0.04, suggesting a practically significant effect size [88]. Several studies mentioned the association of PUSN with our two main variables (BV and loneliness).

First, our study found a link between bullying and excessive use of social networks. This statement is in line with earlier research that has shown an increase in social media use among bullying victims [61,62]. The association of BV with PUSN can be interpreted through the coping mechanism that bullied adolescents use in order to reduce anxiety and distress [74]. Social media leads adolescents to build their online ideal life on social network accounts [75]. In particular during COVID-19 pandemic, the use of social networks was observed [29]. During the pandemic, people in general turned to social media to compensate for social relationships [76]. Moreover, SC may be a factor leading bullied victims to overuse social network. On the one hand, a study showed that bullied children may attribute their peer victimization to feeling inferior to their peers, thus to SC such as not being as cool as others [36], while on the other hand, another study showed that SC is associated with social network use [38,39]. As a result, coping with BV using social media and SC can explain “BV → PUSN” path.

Second, “PUSN → loneliness” path can be explained by both a psychosocial and a biological way. Psychosocially, a significant amount of time and effort devoted to social media use has been shown to interfere with functioning in critical life areas, particularly interpersonal relationships [79]. One study found that Internet use would increase loneliness through social isolation [82]. Biologically, dopamine and endogenous opioids are known to mediate social connection experience [89,90] and may be affected by loneliness [91]. On the other hand, variations in dopamine and opioid peptide levels in the basal ganglia were shown to be implicated in the rewarding experiences of addiction and during the binge and withdrawal stages [92]. In withdrawal phase of addiction, and during the decrease of dopamine function, brain recruits stress neurotransmitters [92]. We suggest that, biologically, the dysregulation of the dopaminergic and opioid system during PUSN may contribute to negative emotions such as loneliness.

### 4.5. Clinical Implications

This study provides valuable insights into the research of the association between BV, PUSN, and loneliness. With the increase in the prevalence of PUSN and loneliness among bullied adolescents, the implementation of public health measures targeted to the factors associated with loneliness should be considered. First, instructors and health care workers have a significant role in supporting youth in this critical stage by evaluating adolescents’ psychological health issues [93]. Routine screening techniques can be established to detect BV, PUSN, and loneliness. Furthermore, prevention and intervention programs for BV and PUSN should be integrated into schools to engage students in activities addressing these problems. At home, parental support and emotional involvement can enhance adolescent’s self-esteem and life satisfaction and protect them from the consequences of psychosocial triggers they face at this critical age [94]. While total restriction of Internet use is not the solution, moderate healthy use should be the goal to attend. Another solution is cognitive behavioral therapy; this type of therapy has been shown to be effective in treating many types of behavioral addictions [93]. Moreover, physical activity can help reduce stress levels and boost self-confidence [95]. In addition, this research found associations between smartphone usage behaviors, BV, and loneliness, suggesting that we may be able to build an early detection system for bullying and loneliness using smartphone usage habits. Finally, there is a need to conduct long-term research on the short and long-term mental health effects of bullying among adolescents, in particular during crisis such as pandemics.

### 4.6. Limitations

For this study, several limitations can be noted. First, social desirability and recall biases may affect participants’ responses. Second, our results cannot be taken for causal associations since our data collection was conducted at a single time point. We emphasize indeed that due to the cross-sectional design, the present estimations of a mediation effect are rather correlational in nature, and the correct causal ordering assumption cannot be tested. In this regard, reverse causality between psychopathology and PUSN has previously been observed, though findings remain inconclusive on this point [96]. To establish causality, further longitudinal studies that establish the temporal order of these two variables, and experimental studies in which the researcher can manipulate the independent variable, are needed to have a stronger indication of the direction of causality of these two variables. We are thus aware that our findings are only preliminary, and we caution readers against interpreting these results causally, until future longitudinal research confirms our findings. Third, the scales used in this study, with the exception of the IBS, have not been validated in Lebanon. Furthermore, due to the self-report nature of the questionnaire used, reporting bias cannot be excluded. For greater validity, future studies might consider collecting bullying- and loneliness-related information from peers, teachers, and parents. A selection bias is present due to the snowball technique followed in sample recruitment. In addition, different confounding variables such as drug use, family interactions, school life satisfaction, etc., might play key roles in the relationship between our variables and their mediating effects should be tested in future studies. Finally, correlational research is important to study the associations between BV, PUSN, and loneliness; however, a longitudinal study is needed to understand how these variables interact over time. Despite these limitations, this is the first to show the association between BV and loneliness among Lebanese adolescents, considering the indirect effect of PUSN.

## 5. Conclusions

Our results show that bullied adolescents have higher rates of loneliness, with an indirect role of problematic social network use. All of these psychological distresses should be taken into consideration by public health authorities, schools, and parents in order to limit the incidence of these problems in youth, especially during times of crisis such as pandemics. Additional research is needed to further clarify the physiological mechanisms underlying the associations between social triggers and loneliness during adolescence. Understanding the neurobiological pathways and brain areas affected by factors related to loneliness may help predict other factors and protect the youth from stressful events that may occur during this critical period.

## Figures and Tables

**Table 1:** Sociodemographic and other characteristics of the participants (N = 379).

Variable	N (%)
Sex	
Male	133 (35.1%)
Female	246 (64.9%)
	**Mean ± SD**
Age (in years) [range: 13–17]	16.07 ± 1.19
Physical activity index	27.78 ± 20.15
Household crowding index (persons/room)	1.26 ± 0.74
Body Mass Index (kg/m^2^)	22.33 ± 3.79
Financial burden [range: 1–10]	4.96 ± 2.80
Negative social comparison [range: 10–50]	20.97 ± 9.73
Addictive consequences [range: 8–40]	19.53 ± 7.46
Loneliness [range: 0–5]	2.01 ± 1.73
Bullying victimization	3.30 ± 5.01

**Table 2:** Bivariate analysis of the continuous variables associated with loneliness.

Variable	L	NSC	AC	BV	Age	PAI	HCI	BMI	FB
Loneliness (L)	1								
Negative social comparison (NSC)	0.52 ***	1							
Addictive consequences (AC)	0.42 ***	0.72 ***	1						
Bullying victimization (BV)	0.27 ***	0.32 ***	0.28 ***	1					
Age	0.14 **	−0.05	−0.01	0.07	1				
Physical activity index (PAI)	−0.06	−0.14 **	−0.16 **	0.09	0.02	1			
Household crowding index (HCI)	0.003	−0.09	−0.09	0.003	0.02	−0.06	1		
Body Mass Index (BMI)	0.03	0.15 **	0.11 *	0.03	0.02	0.03	−0.15 **	1	
Financial burden (FB)	0.25 ***	0.20 ***	0.17 **	0.25 ***	0.14 **	−0.15 **	0.20 ***	0.11 *	1

*r* = Pearson correlation coefficient; * *p* < 0.05; ** *p* < 0.01; *** *p* < 0.001.

**Table 3:** Indirect effect analyses results, taking bullying victimization as the independent variable, the problematic use of social network subscales as mediators and loneliness as the dependent variable.

	Direct Effect			Indirect Effect		
	Beta	SE	*p*	Beta	Boot SE	Boot CI
Negative social comparison	0.10	0.02	<0.001	0.04	0.02	0.01–0.07 *
Addictive consequences	0.06	0.02	<0.001	0.03	0.01	0.02–0.05 *

* indicates significant indirect effect.

## Data Availability

The authors do not have the right to share any data information as per the ethics committee rules and regulations.
